# Antiestrogens: structure-activity relationships and use in breast cancer treatment

**DOI:** 10.1530/JME-16-0024

**Published:** 2016-12-03

**Authors:** T Traboulsi, M El Ezzy, J L Gleason, S Mader

**Affiliations:** 1 Institute for Research in Immunology and CancerUniversité de Montréal, Montréal, Québec, Canada; 2 Department of Biochemistry and Molecular MedicineUniversité de Montréal, Montréal, Québec, Canada; 3 Department of ChemistryMcGill University, Montréal, Québec, Canada

**Keywords:** breast cancer, estrogen receptors, antiestrogens, SUMOylation, ubiquitination

## Abstract

About 70% of breast tumors express estrogen receptor alpha (ERα), which mediates the proliferative effects of estrogens on breast epithelial cells, and are candidates for treatment with antiestrogens, steroidal or non-steroidal molecules designed to compete with estrogens and antagonize ERs. The variable patterns of activity of antiestrogens (AEs) in estrogen target tissues and the lack of systematic cross-resistance between different types of molecules have provided evidence for different mechanisms of action. AEs are typically classified as selective estrogen receptor modulators (SERMs), which display tissue-specific partial agonist activity (e.g. tamoxifen and raloxifene), or as pure AEs (e.g. fulvestrant), which enhance ERα post-translational modification by ubiquitin-like molecules and accelerate its proteasomal degradation. Characterization of second- and third-generation AEs, however, suggests the induction of diverse ERα structural conformations, resulting in variable degrees of receptor downregulation and different patterns of systemic properties in animal models and in the clinic.

## Introduction

**Figure 1:**
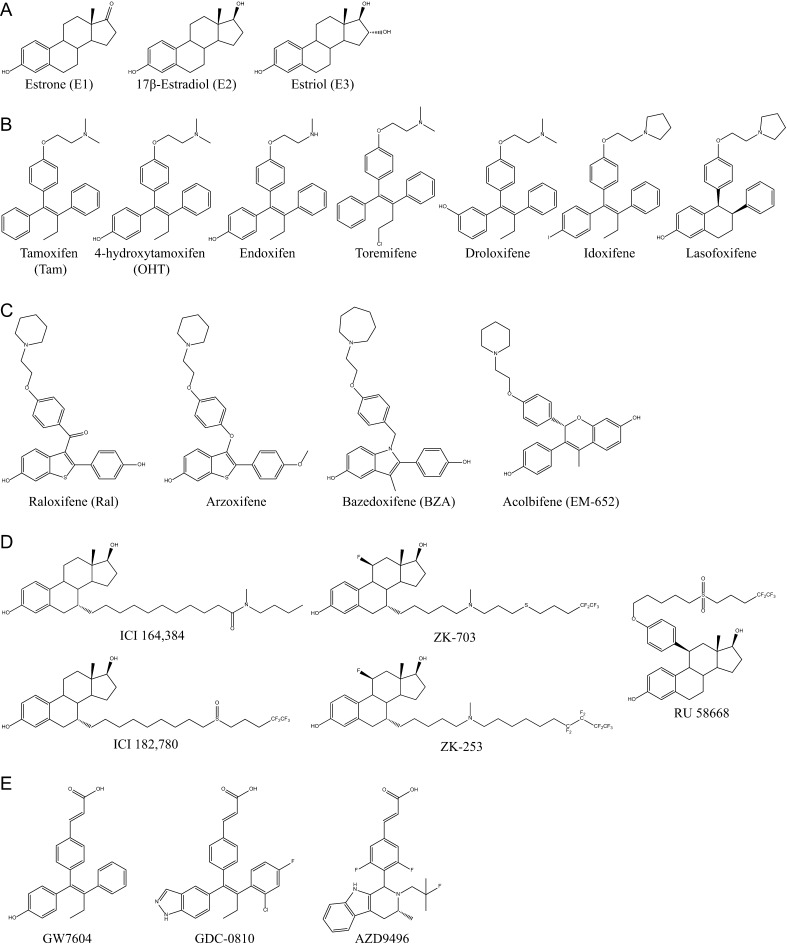
Chemical structures of estrogen receptor agonists and antagonists. (A) The three most abundant circulating estrogens: estrone, 17β-estradiol and estriol. (B) Tamoxifen and its active derivatives, 4-hydroxytamoxifen and endoxifen, as well as tamoxifen-derived SERMs. (C) Antiestrogens with different steroid-like backbones and a side chain containing a tertiary amine: SERM raloxifene and related compounds arzoxifene and bazedoxifene, as well as acolbifene. (D) Pure antiestrogens with steroid backbones and long side chains. (E) SERDs with steroid-like backbones and a side chain containing an acrylic acid.

Estrogen receptors (ERα and ERβ) control a range of physiological processes regulating the development and function of the female reproductive system as well as the maintenance of bone mass, and play protective roles in the cardiovascular and central nervous systems. ERs are also implicated in related pathologies such as breast and uterine cancers, osteoporosis and cardiovascular diseases (Nilsson *et al*. 2001, Deroo & Korach 2006, Jia *et al*. 2015). Their roles in cancer development have led to the development and clinical use of small synthetic molecules that block either estrogen production (aromatase inhibitors) or estrogenic signaling (antiestrogens, AEs). AEs are steroids or steroid mimics that compete with endogenous estrogens (Fig. 1A) for binding to ERs and modify their activity as ligand-dependent transcriptional regulators (Hall *et al*. 2001, Ascenzi *et al*. 2006). However, some AEs, including tamoxifen and raloxifene, have complex patterns of activity in estrogen-responsive tissues, acting as so-called selective estrogen receptor modulators (SERMs). For instance, tamoxifen displays mostly antagonist activity in breast but has partial agonist activity in uterus and bones (Ward *et al*. 1993, Klotz *et al*. 2000, O’Regan & Jordan 2002).

On the other hand, fulvestrant and related compounds are devoid of partial agonist activity and behave as pure AEs. The lack of systematic cross-resistance to pure AEs observed in tumors that have progressed under treatment with tamoxifen, or in cell lines that have gained the capacity to grow in the presence of SERMs, suggested that pure AEs and SERMs have different mechanisms of action (Howell 2006, Ali *et al*. 2011). Properties characteristic of pure AEs include induction of accelerated turnover of ERα via increased proteasomal degradation; hence, their designation as selective estrogen receptor downregulators (SERDs) (McDonnell *et al*. 2015). Although fulvestrant has failed to demonstrate improved responses in first-line treatment compared with SERMs or aromatase inhibitors (Howell *et al*. 2004*a*), a better understanding of the mechanisms of action of pure AEs is important in view of the development and current clinical testing of several new SERDs with improved oral bioavailability compared with fulvestrant (Callis *et al*. 2015, McDonnell *et al*. 2015). This review focuses on what is known and what remains to be determined about the mechanisms of action of AEs, with an emphasis on properties specific to pure AEs.

## SERMs vs SERDs: two separate classes of antiestrogens?

### Tamoxifen and next-generation SERMs

Selective ER modulators (SERMs) (e.g., tamoxifen, raloxifene and analogues, Fig. 1B and C) have earned their name due to their tissue- and gene-specific activities. Tamoxifen, the first clinically approved SERM and the standard of care for the adjuvant treatment of all stages of primary breast tumors to this day (Jordan 2004, Ali *et al*. 2011, Martinkovich *et al*. 2014), has antagonist effects on breast cancer cell proliferation but has an estrogen-like action in bone in patients and in animal models, where it helps maintain bone mineral density in postmenopausal women, and has favorable agonist effects on lipid profiles (Turner *et al*. 1988, Ward *et al*. 1993, Love *et al*. 1994*a*,*b*). In addition, tamoxifen and its active metabolite 4-hydroxytamoxifen have marked estrogenic activity in the uterus of ovariectomized rats or mice and are associated with an increased risk of endometrial cancer in the clinic (Martin & Middleton 1978, Davies *et al*. 1979). Increases in mouse uterine wet weight induced by tamoxifen were dependent on the expression of ERα in the uterus (Korach 1994). In addition, although tamoxifen treatment has proven effective in reducing the risk of breast tumor progression, resistance to tamoxifen develops in a significant proportion of primary tumors and in most patients with metastatic cancer, without loss of ERα expression in the majority of cases (Jordan 2004, Musgrove & Sutherland 2009). Observation of remissions after tamoxifen withdrawal or switch to aromatase inhibitors or pure AEs has suggested that ER signaling remains active in the presence of tamoxifen in some tamoxifen-resistant breast tumors (Ali *et al*. 2011, McDonnell *et al*. 2015).

Tamoxifen analogues (Fig. 1B) have been developed to increase treatment efficacy and decrease the negative side effects of tamoxifen, including the increase in the incidence of endometrial cancer and thromboembolic events and the generation of DNA adducts (see Martinkovich *et al*. 2014 for an in-depth review of the pharmacological properties of tamoxifen analogues). For example, toremifene (Fig. 1B) is a chloride derivative of tamoxifen that has been reported to have lower estrogenic and genotoxic effects than tamoxifen. Similarly, tamoxifen derivatives droloxifene (3-hydroxytamoxifen), which has increased affinity for ERα but a reduced half-life, and idoxifene, which is metabolized more slowly than tamoxifen due to the addition of an iodine at position 4 and has a modified side chain with a pyrrolidino group (Fig. 1B), were both found to have decreased uterotrophic activity. Lasofoxifene, a tamoxifen analogue with a modified polycyclic core structure and a side chain similar to that of idoxifene (Fig. 1B), is an antagonist in both breast and uterus. All these compounds possess a strong agonist activity in bones. However, these drugs are neither more efficacious than tamoxifen for breast cancer treatment nor do they circumvent resistance to tamoxifen in patients (Howell 2006, Ali *et al*. 2011, Martinkovich *et al*. 2014).

Raloxifene, a SERM with a benzothiophene backbone (Fig. 1C) that is prescribed for prevention of osteoporosis and associated with favorable agonist-like action on lipid metabolism, has only low activity in the uterus of ovariectomized rodents (Black *et al*. 1994). Raloxifene was shown to retain 76% of the effectiveness of tamoxifen at reducing invasive breast cancer incidence with a significantly lower incidence of endometrial cancer in the Study of Tamoxifen and Raloxifen (STAR) prevention trial (Vogel *et al*. 2010), but is not effective in patients resistant to tamoxifen (Ali *et al*. 2011, Martinkovich *et al*. 2014). Further, the raloxifene analogue arzoxifene (Fig. 1C), despite being more potent than tamoxifen or 4-hydroxytamoxifen on inhibition of human mammary carcinoma cell proliferation and on decreasing rat uterine wet weight (Palkowitz *et al*. 1997, Suh *et al*. 2001), was not as efficacious as tamoxifen in a comparative phase III clinical trial (Deshmane *et al*. 2007) and was partially cross-resistant with tamoxifen in xenograft models (Schafer *et al*. 2001).

### Fulvestrant and other SERDs with long side chains

Another class of AEs developed to minimize partial agonist activity and address resistance issues (Wakeling 1993) includes steroidal compounds with long side chains such as ICI 164,384, ICI 182,780 (fulvestrant/Faslodex) and RU 58,668 (Fig. 1D). These compounds were initially referred to as pure AEs due to their lack of partial agonist activity in pre-clinical models including in breast and endometrial cell lines (Bowler *et al*. 1989, Wakeling *et al*. 1991, Van de Velde *et al*. 1994, Barsalou *et al*. 1998). Moreover, ICI 182,780 was able to suppress the agonist activity of estradiol and tamoxifen in the uterus of ovariectomized rodents (Wakeling *et al*. 1991). ICI 182,780 also does not have an agonist effect on bone cells *in vitro* and in animal models (Gallagher *et al*. 1993, Ciana *et al*. 2001, Park 2013). Although ICI 182,780 is not orally bioavailable, its subcutaneous injection (5 mg/week) suppressed the growth of MCF-7 xenografts in mice longer than tamoxifen (500 μg/day s.c.) (Osborne *et al*. 1995). Further, RU 58,668 can cause long-term regression of MCF-7 xenografts (Van de Velde *et al*. 1994). Importantly, cross-resistance between ICI 182,780 and tamoxifen was not observed in cultured cell lines or in xenograft models (Hu *et al*. 1993, Lykkesfeldt *et al*. 1994, 1995); further, fulvestrant was comparable to aromatase inhibitors in clinical efficacy in postmenopausal women having progressed on tamoxifen therapy (Howell *et al*. 2002, Osborne *et al*. 2002). These data suggested that pure AEs and SERMs act via different molecular mechanisms. Indeed, although 4-hydroxytamoxifen increases overall ERα protein levels, pure AEs accelerate ERα turnover through the ubiquitin–proteasome pathway in ERα-positive breast cancer cells and in extracts of rodent uterine tissues, hence, their designation as SERDs (Gibson *et al*. 1991, Dauvois *et al*. 1992, El Khissiin & Leclercq 1999, Wijayaratne & McDonnell 2001). However, despite the pure antiestrogenic character of fulvestrant, it did not compare advantageously with tamoxifen when used as a first-line therapy for advanced or metastatic breast cancer (Howell *et al*. 2004*a*, Howell 2006). The poor pharmacokinetic properties of ICI 182,780 may limit its effectiveness in the clinic; indeed, increasing monthly intra-muscular injections of fulvestrant from 250 mg to 500 mg led to significant gains in overall survival in metastatic patients having recurred or progressed after prior endocrine therapy in the CONFIRM study and resulted in subsequent adoption of this regimen in the clinic (Di Leo *et al*. 2010, 2014, Robertson *et al*. 2014). Further development of pure AEs has focused on gains in affinity and oral bioavailability. For instance, fulvestrant analogues ZK-703 and ZK-253 (Fig. 1D) were shown to inhibit growth of ER+ xenografts, including tamoxifen-resistant tumors, more efficiently than ICI 182,780; interestingly, compound ZK-253 demonstrated increased oral bioavailability in these models (Hoffmann *et al*. 2004).

### SERM derivatives with SERD activity

Interestingly, compounds derived from tamoxifen such as GW7604 and analogues (Fig. 1E) can also induce ERα degradation (Wijayaratne *et al*. 1999, Bentrem *et al*. 2001) and may prove to be promising clinical candidates as they have better oral bioavailability than ICI 182,780 (McDonnell *et al*. 2015). Of note, GDC-0810 induced degradation of ERα with similar potency and efficacy as ICI 182,780 and was effective at suppressing growth of both tamoxifen-sensitive and -resistant xenografts (Lai *et al*. 2015). GDC-0810 and a structural analogue (AZD9496) are undergoing evaluation in currently accruing clinical trials (see https://clinicaltrials.gov/ct2/results?term=GDC-0810&Search=Search; accessed Aug. 15, 2016). In addition, although raloxifene induces a slight increase in ERα turnover, raloxifene-related bazedoxifene (Fig. 1C) is more efficacious in this respect, correlating with fuller antiestrogenic properties. Bazedoxifene is indeed more effective than other SERMs (tamoxifen, raloxifene and lasofoxifene) at inhibiting gene expression in MCF-7 cells and can inhibit tamoxifen-resistant xenograft growth (Wardell *et al*. 2013). Bazedoxifene is currently prescribed for the prevention and treatment of osteoporosis, as bazedoxifene and raloxifene had similar impacts on bone mineral density and lipid profiles in a 24-month randomized clinical study (Miller *et al*. 2008) and on ER signaling in bone (Rando *et al*. 2010). A phase I/II clinical trial currently investigates the combination of the CDK4/6 inhibitor Palbociclib and bazedoxifene in stage IV breast cancer patients (see https://clinicaltrials.gov/ct2/show/Nbib2448771; accessed Aug. 15, 2016). Similarly, although EM-800 and its active metabolite EM-652 (acolbifene, Fig. 1C) are SERMs, displaying agonist activity in bone and on lipid metabolism but limited estrogenic activity in the uterus (Howell *et al*. 2004*b*), EM-800 induces accelerated turnover of ERα compared to tamoxifen, albeit to a lesser extent than raloxifene (Wittmann *et al*. 2007).

Therefore, it appears that AEs present a spectrum of SERD activity, with tamoxifen at one end being devoid of ERα down-regulating capacity, whereas some SERM analogues reduce ERα protein levels to variable levels (EM-652, raloxifene, bazedoxifene, GW7604 and GDC-0810). Finally, pure AEs with long side chains are associated with strong SERD activity (ICI 164,384, ICI 182,780, ZK-253, ZK-703 and RU 58,668).

## Molecular determinants of antiestrogenicity

### Structural basis for AF2 transcriptional activity

**Figure 2:**
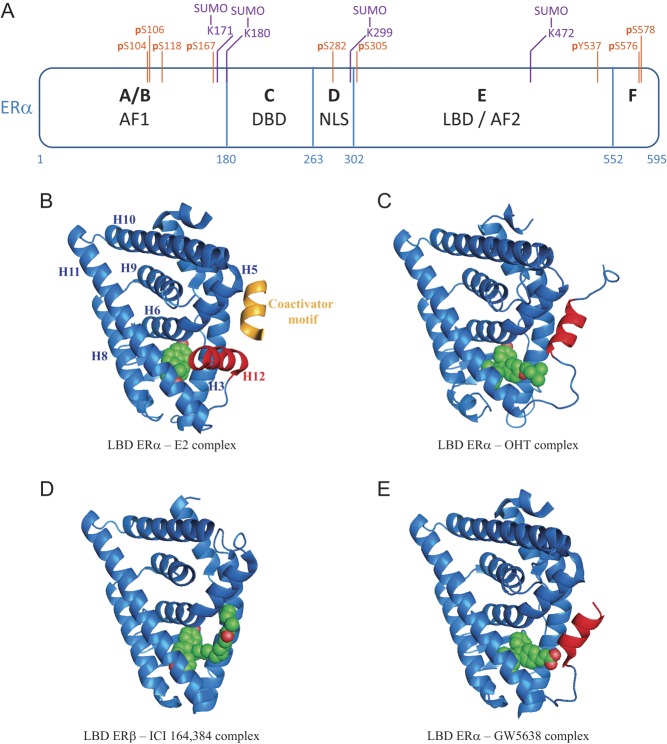
ERα structure, post-translational modifications and conformational changes induced by different ligands. (A) Schematic representation of ERα structure. AF1/AF2: activation function 1/2; DBD: DNA-Binding Domain; NLS: Nuclear Localization Signal; LBD: Ligand-Binding Domain. SUMOylation sites identified by mass spectrometry in the presence of ICI 182,780 are indicated in purple. Residues phosphorylated in the presence of antiestrogens or implicated in the modulation of sensitivity to antiestrogen treatment are indicated in orange. (B) LBD ERα – estradiol (E2) – TIF2 NR box 3 complex (Warnmark *et al*. 2002); (C) LBD ERα – 4-hydroxytamoxifen complex (OHT) (Shiau *et al*. 1998); (D) LBD ERβ – ICI 164,384 complex (Pike *et al*. 2001); (E) LBD ERα – GW5638 complex (Wu *et al*. 2005). Representations were generated using PyMOL. Helix 12 is highlighted in red and each ligand is shown in green. The α-helical TIF2 coactivator motif is shown in gold.

ERs share with other nuclear receptors (NRs) a common structure with a central DNA-binding domain (DBD) flanked by two transcriptional activation function domains, AF1 and AF2, the latter overlapping with the ligand-binding domain (LBD) (Fig. 2A). Ligand binding regulates NR conformation, nuclear localization, binding to specific response elements and recruitment of coactivators/corepressors (Moras & Gronemeyer 1998, Weatherman *et al*. 1999, Aranda & Pascual 2001, Sanchez *et al*. 2002, White *et al*. 2004). Although unstable in the absence of ligand, the LBD can be crystallized in the presence of agonists (estradiol, E2), revealing folding into an α-helical sandwich structure characteristic of the nuclear receptor superfamily. The ligand-binding pocket is formed within the hydrophobic core of the LBD below the central layer of helices (Fig. 2B) and is lined by hydrophobic residues from H3, H6, H8, H11, H12 and the S1/S2 hairpin (Brzozowski *et al*. 1997). Charged amino acids stabilize the binding of agonists and antagonists by interacting with hydroxyl groups located at either end of the estrogenic steroidal backbone (Glu353, Arg394 and His524 in hERα; Glu260, Arg301 and His430 in rERβ). Agonist (E2) binding stabilizes a conformation of the ligand-binding domain where H12 folds tightly back on top of the ligand-binding pocket (Fig. 2B), positioning a set of amino acids (Asp538, Leu539, Glu542 and Met543) adequately for coactivator peptide interaction (Brzozowski *et al*. 1997, Shiau *et al*. 1998, Warnmark *et al*. 2002).

### Impact of SERMs on AF2 activity

AEs are steroid or steroid-like (e.g. triphenylethylene or benzothiophene backbones of tamoxifen or raloxifene, respectively) molecules that bind to the ERα LBD in a manner similar to estradiol. Bulky side chains attached at positions 7α or 11β of a steroid core or at equivalent positions on a steroid-like skeleton are responsible for antiestrogenicity (Jordan 2004). They project out of the ligand-binding cavity between helices 3 and 11 and prevent the positioning of H12 over the ligand-binding cavity via steric clashes. This is achieved through different structural rearrangements depending on the length and composition of the AE side chain.

**Figure 3:**
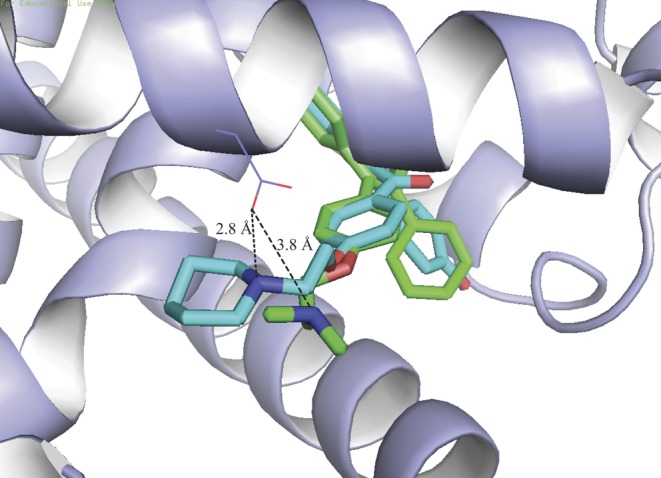
Role of Asp351 in the different activity of tamoxifen and raloxifene. Overlay of X-ray structures of 4-hydroxytamoxifen (green) and raloxifene (aqua) bound to ERα (data from Shiau *et al.* (1998) and Brzozowski *et al.* (1997), respectively). The distance from Asp351 to the dimethylamine in 4-hydroxytamoxifen (3.8 Å) is 1.0 Å longer than to the piperidine in raloxifene.

Several SERMs including tamoxifen and raloxifene and analogues (Fig. 1B and C) contain alkylaminoethoxy side chains with different tertiary amine substituents. Steric clashes involving the dimethylamino group of tamoxifen or the piperidyl group of raloxifene favor the repositioning of H12 to the coactivator-binding groove (Brzozowski *et al*. 1997, Shiau *et al*. 1998) (Fig. 2C 4-hydroxytamoxifen, OHT). Hydrophobic amino acids in H12 (Leu540, Met543 and Leu544) interact with the coactivator-binding groove in a manner similar to amphipathic coactivator LXXLL motifs (Shiau *et al*. 1998, Pike *et al*. 1999). Replacing the nitrogen in the raloxifene side chain with a carbon or a non-basic nitrogen atom abolished the antagonist activity of raloxifene derivatives in uterine wet weight assays (Grese *et al*. 1997) and induced ER-dependent transcription in stably transfected MDA-MB-231 cells (Liu *et al*. 2002). Crystallographic studies have revealed that the tertiary amine of raloxifene forms a hydrogen bond with Asp351 in H3 of the ERα LBD (Fig. 3) (Brzozowski *et al*. 1997). Mutating Asp351 to Glu converted raloxifene, which behaves as a pure antagonist in transiently transfected HepG2 cells, into a partial agonist resembling tamoxifen (Dayan *et al*. 2006). Interaction of the tertiary amine with Asp351 appears weaker in tamoxifen than that in raloxifene (3.8 vs 2.8 Å, Fig. 3) and the D351E mutation had little effect on the partial agonist activity of tamoxifen in HepG2 cells. Exchanging the tertiary amine group in tamoxifen for that of idoxifene led to loss of partial agonism with wt ERα, suggesting optimized interaction with Asp351. Conversely, partial agonism of this molecule was restored to levels comparable with those of tamoxifen by the D351E mutation, similar to observations with raloxifene (Dayan *et al*. 2006). Mutation D351G abrogated induction of expression of the estrogen target gene *TGFA* by tamoxifen in transfected MDA-MB-231 cells (MacGregor Schafer *et al*. 2000), and mutation D351A abolished partial activity of ERα on a reporter gene in the presence of tamoxifen in HepG2 cells (Dayan *et al*. 2006), consistent with a role of Asp351 in mediating the partial agonist activity of SERMs in the absence of optimal interaction with their side chain tertiary amines.

### Impact of pure AEs on AF2 activity

**Figure 4:**
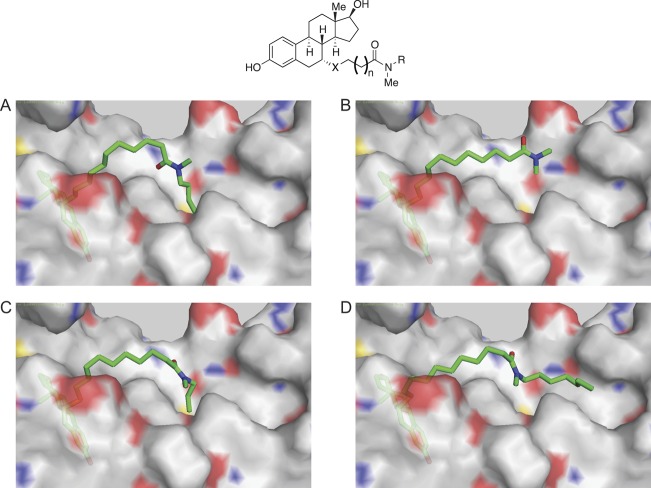
Models of ICI 164,384 and derivatives bound to ERβ. (A) ICI 164,384 (X = CH_2_, *n* = 9, R = C_4_H_9_); (B) a 13-atom side chain (X = S, *n* = 8, R = CH_3_); (C) a 15-atom side chain (X = S, *n* = 8, R = C_3_H_7_); (D) a 19-atom side chain (X = S, *n* = 8, R = C_7_H_15_); docking was performed using the Glide software as previously described (Hilmi *et al*. 2012).

First-generation pure AEs such as fulvestrant have longer side chains than SERMs (Fig. 1D). A crystal structure of ICI 164,384 with the rat ERβ LBD reveals that the long side chain at position 7α exits the ligand-binding cavity in a manner similar to that of the SERM side chains, but bends by 90 degrees at its fifth carbon, hugging the surface of the LBD and interacting with the coactivator-binding groove (Pike *et al*. 2001) (Fig. 2D). The terminal hydrophobic n-butyl group of ICI 164,384 fits into a pocket formed by the side chains of Leu261, Met264, Ile265 and Leu286 in the coactivator-binding groove of rat ERβ (Leu354, Met357, Ile358 and Leu379 in human ERα). This interaction displaces H12 from its position in the binding groove observed in structures with 4-hydroxytamoxifen (OHT) and raloxifene (Fig. 2C) (Brzozowski *et al*. 1997, Shiau *et al*. 1998). H12 is disordered in the crystal structure with ICI 164,384, suggestive of high mobility (Pike *et al*. 2001). Although this structure was obtained with ERβ, and binding of ICI 164,384 does not induce accelerated degradation of this receptor (Peekhaus *et al*. 2004), the relevance of side chain interaction with the coactivator-binding groove of ERα (lined with amino acids conserved with ERβ) for pure antiestrogenicity has been supported by the analysis of ICI 164,384 derivatives with variable side chain lengths. Pure antiestrogenicity was optimal with side chain lengths of 15–19 atoms in a reporter assay in HepG2 cells transiently transfected with ERα, whereas the addition of shorter side chains (13 or 14 carbons side chains) resulted either in agonist or SERM activity (Hilmi *et al*. 2012, Hoffman *et al*. 2012). Molecular modeling of these ICI 164,384 derivatives in complex with the ERβ LBD suggests that pure antiestrogenicity is associated with chain lengths long enough to reach the coactivator-binding groove (Fig. 4 and Video 1). This is also compatible with the observed importance of the hydrophobicity of the terminal substituents for pure antiestrogenicity in steroidal derivatives. ICI 182,780, with a penta fluoropentyl terminal substituent, showed increased potency and efficacy in growth inhibition compared with ICI 164,384 in both cell and animal models of human breast cancer (Wakeling *et al*. 1991).

Video 1 Animation of the models of ICI 164,384 derivatives with 13 and 15-atom side chains bound to ERβ (corresponding to Fig. 4B and C). View Video 1 at http://movie-usa.glencoesoftware.com/video/10.1530/JME-16-0024/video-1.
Download Video 1

The side chain of ICI 164,384 creates steric clashes with H12 in the agonist conformation at amino acids Leu540 and Met543. Furthermore, it leads to steric clashes with Leu536, and to a lesser extent Leu540, when H12 is positioned in the coactivator-binding groove. Ala mutation of these residues increased transcriptional activity of ERα in the presence of pure AEs (Mahfoudi *et al*. 1995, Norris *et al*. 1998, Lupien *et al*. 2007, Arao *et al*. 2011), presumably by reducing steric clashes with H12.

Although the antiestrogenicity of ICI 182,780 is not affected by Asp351 mutations (Dayan *et al*. 2006), introduction of a tertiary amine in the ICI 182,780 side chain was associated with improved efficacy of compounds ZK-703 and ZK-253 at preventing growth of mouse xenografts from estrogen-sensitive and tamoxifen-resistant breast cancer lines (Hoffmann *et al*. 2004). Whether interaction with Asp351 is important for the improved performance of these compounds remains however to be assessed.

### Molecular basis for SERD activity in SERM derivatives

Alterations in the shorter side chains of SERM derivatives have also been observed to result in partial or full SERD activity. Bazedoxifene’s overall structure is similar to that of raloxifene and differs by having a bulkier heterocyclic amine ring (azepane instead of piperidine ring, Fig. 1C), which may result in increased steric hindrance with H12. In addition, GW5638 (pro-drug of GW7604) is a tamoxifen analogue in which the dimethylaminoethoxy group is replaced by an acrylic acid side chain (Fig. 1E). The carboxylate group in the GW5638 side chain, in its protonated state, forms hydrogen bonds with Asp351 and the peptidic backbone of H12. This results in a distinct conformation of H12 (Fig. 2E) with relocation of the side chains of hydrophobic residues (Leu536, Leu539, Leu540 and Met543) toward the protein exterior, increasing the exposed hydrophobic surface of H12 compared with the ERα – 4-hydroxytamoxifen structure while preserving interaction in the coactivator-binding groove (Wu *et al*. 2005).

Thus, pure antiestrogenicity appears associated with exposure of hydrophobic amino acids of H12 to the solvent irrespective of the precise positioning of H12 in crystal structures (occupancy of the coactivator-binding groove in GW7604, but not in ICI 164,384). How these structural features result in altered protein–protein interactions and in a decreased stability of ERα is still imperfectly understood.

## Impact of AE-induced ERα conformation on cofactor recruitment and transcriptional activation

### Gene expression profiles of SERMs and SERDs in breast and uterine cancer cell models

The characterization of gene expression profiles in the presence of AEs in breast and uterine cancer cells has indicated that SERMs display partial agonist activity in a gene-specific manner, while SERDs achieve a more complete inhibition of estrogen signaling. Indeed, tamoxifen regulates transcription of subsets of estrogen target genes in endometrial carcinoma cell lines (Ishikawa, ECC-1) and also in ER+ breast cancer cell lines (MCF-7, ZR-75-1), in addition to altering expression of sets of genes apparently not regulated by estradiol via mechanisms that remain to be clarified (Shang & Brown 2002, Frasor *et al*. 2004, Scafoglio *et al*. 2006, Chang *et al*. 2010, Wardell *et al*. 2012, Tamm-Rosenstein *et al*. 2013). ICI 182,780 functions as a pure antagonist for genes partially activated by SERMs in MCF-7 cells, whereas raloxifene has an intermediate profile, its agonist activity mostly overlapping with that of tamoxifen. On the other hand, bazedoxifene exhibits a SERD-like profile (Frasor *et al*. 2004, Wardell *et al*. 2013). ChIP-seq experiments in MCF-7 cells have shown binding of ERα to a significant number of estrogen target sites after 1 h of ICI 182,780 treatment (Welboren *et al*. 2009); in contrast, association of ERα with DNA was not observed 3 h after addition of ICI 182,780 in another study (Reid *et al*. 2003), possibly due to receptor degradation. It will be of interest to examine whether release of ERα from DNA is a general property of SERDs and correlates with the degradation of the receptor in the presence of these ligands, or with earlier events such as protein modification and/or altered cofactor recruitment.

### Impact of SERMs and SERDs on coactivator recruitment to ERα

ERs recruit a plethora of cofactors in an agonist-dependent manner via both their N-terminal and C-terminal activation function regions (AF1 and AF2 respectively), including histone modifiers, chromatin remodeling complexes and components of the transcriptional machinery (Smith & O’Malley 2004, Hall & McDonnell 2005); (see also a list of known nuclear receptor coregulators at https://www.nursa.org/nursa/molecules/index.jsf, accessed on Aug. 15, 2016). The various ERα LBD conformations induced by different AEs affect protein–protein interaction interfaces (Wardell *et al*. 2013) and result in altered recruitment of cofactors both in solution and on DNA.

Among the coactivators interacting directly or indirectly with AF2 of the estradiol-bound ERα are the histone acetyl transferases NCOA1/2/3 (SRC-1/2/3), CBP/p300 and the histone methyl transferases CARM1, PRMT1 (Smith & O’Malley 2004, Hall & McDonnell 2005, Johnson & O’Malley 2012). In endometrial Ishikawa and ECC-1 cell lines, NCOA1 is recruited selectively to promoters of genes stimulated by tamoxifen, but not raloxifene; repressing NCOA1 expression in Ishikawa cells inhibits the partial agonist activity of tamoxifen on those target genes (Shang & Brown 2002). Conversely, overexpressing NCOA1 in MCF-7 cells confers agonist activity to tamoxifen on genes it otherwise antagonizes, suggesting that differential expression of NCOA1 in breast and uterine cells underlies tissue-specific transcriptional regulation by tamoxifen (Shang & Brown 2002). In addition, overexpressing the coactivators NCOA2 and p300 in ERα-transfected HeLa cells strongly increased the partial agonism of tamoxifen on an ERE-TK-Luc reporter vector, had a moderate effect for raloxifene and barely increased reporter vector activity in the presence of ICI 182,780 (Webb *et al*. 2003), suggesting that several coactivators may contribute to the partial agonist activity of SERMs in a cell- and gene-specific manner. Finally, NCOA3 (AIB1) is amplified in 11% of breast tumors and is associated with a worse prognosis in ER+, but also ER− tumors (Burandt *et al*. 2013); its tumorigenic potential may therefore result from a role as coactivator of other transcription factors, such as E2F1 (Louie *et al*. 2004).

The partial agonist activity of tamoxifen, and to a lower degree of raloxifene, has been linked with activity of the ligand-independent AF1 function of ERα (Fig. 2A) in different cell and promoter contexts (Berry *et al*. 1990, Tzukerman *et al*. 1994, Onate *et al*. 1998, Webb *et al*. 1998, 2003, Benecke *et al*. 2000, Metivier *et al*. 2001). For instance, the AF1 domain of ERα was necessary to observe agonist effects of tamoxifen in a human endometrial cancer cell line (HEC1 cells) (McInerney *et al*. 1998). Similarly, swapping the AF1 domain of ERα with the corresponding region in ERβ abrogated ERα transcriptional activity in the presence of tamoxifen in U2OS cells, supporting the role of this region in the partial agonism of tamoxifen (Zwart *et al*. 2010). These observations can be correlated to the capacity of ERα, but not of ERβ, to recruit NCOA1 via its AF1 region in the presence of tamoxifen (Webb *et al*. 1998, Merot *et al*. 2004). It remains unclear whether ERα can also recruit other cofactors in the presence of tamoxifen via its AF1 region, such as the p68 RNA helicase and the RNA molecule SRA (Lanz *et al*. 1999, Janknecht 2010), and how cofactor recruitment via AF1 is enabled by the specific conformation of AF2 in tamoxifen-liganded ERα (Arao *et al*. 2015).

### Impact of SERMs and SERDs on corepressor recruitment

In the presence of tamoxifen, ERα recruits the corepressors NCOR1 (N-CoR) and NCOR2 (SMRT) at repressed estrogen target genes in MCF-7 cells, but not at genes upregulated by tamoxifen in Ishikawa cells; siRNA knockdown of these corepressors increases ER target gene expression and MCF-7 cell proliferation in the presence of tamoxifen *in vitro* and in xenograft models (Lavinsky *et al*. 1998, Shang & Brown 2002, Keeton & Brown 2005). In addition, overexpression of corepressor NCOR2 suppresses the partial agonist activity of tamoxifen in HepG2 cells (Smith *et al*. 1997). ChIP time-course experiments linked the recruitment of NCOR1/HDAC3 and the NuRD/HDAC1 complexes by tamoxifen-bound ERα with subsequent hypoacetylation of histones and loss of RNA Polymerase II binding at the *TFF1* and *MYC* promoters in MCF-7 cells (Liu & Bagchi 2004).

ICI 182,780-bound ERα can recruit the C-terminal fragment of NCOR1 more efficiently than with raloxifene or tamoxifen, as shown by immunoprecipitation experiments in transfected HeLa cells (Webb *et al*. 2003). However, the exact mechanisms of corepressor recruitment in the presence of SERMs and SERDs remain to be determined. Co-crystallization of a corepressor-NR box (CoRNR box, consensus LXXXIXXXL) peptide with the ERα LBD in the presence of raloxifene was only possible upon deletion of H12 (Heldring *et al*. 2007). In this structure, the CoRNR^ER^ peptide occupies the AF2 groove between H3 and H5, the N-terminus of the peptide being packed against the raloxifene side chain. Whether the different conformations of H12 in SERD- vs SERM-bound ERα are responsible for the increased recruitment of corepressors in the presence of SERDs needs to be investigated. Of note, the N-terminal receptor-interacting domain (nRID) of the corepressors NCOR1 and NCOR2 was also shown to interact with ERα via its DBD (Varlakhanova *et al*. 2010), although this interaction did not appear to be ligand regulated.

### Existence of SERM/SERD-specific cofactors

Characterization of proteins interacting with a TAP-tagged version of ERα in MCF-7 cells indicated that the majority of interactors are ligand-specific, the interactomes of ERα bound to raloxifene and tamoxifen overlapping only partially with each other and with that of ERα bound to estradiol and being distinct from that obtained in the presence of ICI 182,780 (Cirillo *et al*. 2013). In addition, ICI 182,780 was shown to selectively induce interaction of ERα, but not ERβ, with luminal cytokeratins CK8/CK18, a property that correlated with ERα insolubility and increased turnover (Long & Nephew 2006, Long *et al*. 2010). Finally, selective recruitment of Ubi and SUMO E3 ligases in the presence of SERDs is likely in view of the patterns of receptor modification induced by SERD binding (see below). The interaction profiles of SERMs with partial SERD activity remain to be investigated, and it will be of interest to determine whether these molecules elicit interactions with some of the ICI 182,780-specific ERα interactors.

### Parameters affecting cofactor recruitment by ERs in the presence of AEs

Although cofactor recruitment is primarily determined by the conformation of the ER LBD induced by ligand binding, the relative expression levels of coactivators and corepressors in different tissues likely account for tissue-specific partial agonist activity (see above). In addition, variation in cofactor expression levels during tumorigenesis may contribute to resistance to AEs (Osborne *et al*. 2003, Su *et al*. 2008). Promoter context is expected to affect partial agonist activity in a gene-specific manner either due to cofactor interaction with other DNA-bound transcription factors or to allosteric effects of the DNA sequence on the receptor conformation (Smith & O’Malley 2004, Johnson & O’Malley 2012). In addition, the relative expression levels of ERα and ERβ, the activity of signaling pathways leading to post-translational modifications of the receptors and/or their coregulators, and the extent of ERα downregulation may all contribute to the specific activity profiles of AEs (Smith & O’Malley 2004, Martinkovich *et al*. 2014).

## Role of post-translational modifications of ERα by Ubi-like molecules in pure antiestrogenicity

Post-translational modifications (PTMs) targeting ERs as well as their cofactors in response to ligand binding likely play a role in modulating cofactor recruitment. Improvements in mass spectrometry have allowed the identification of PTM sites throughout ERα, including phosphorylation, methylation and acetylation. For example, phosphorylation of Ser104, 106 and 118 in the AF1 region and of Ser305 in the E region might be involved in resistance to tamoxifen (Le Romancer *et al*. 2011 and refs within; Fig. 2A). Ser104, 106 and 118 are also phosphorylated in the presence of pure AEs (Ali *et al*. 1993, Thomas *et al*. 2008), but the possible impact of these modifications on transcriptional downregulation by SERDs remains to be investigated. In addition, dephosphorylation of Tyr537 (Fig. 2A) by the H1 protein-tyrosine phosphatase was observed to sensitize MCF-7 breast cancer cells to both SERMs and ICI 182,780 (Suresh *et al*. 2014). Finally, phosphoresidues pS167, pS282, pS576 and pS578 were detected in the presence of ICI 182,780 by mass spectrometry (Hilmi *et al*. 2012) (Fig. 2A), but their function is currently unknown. Other types of modifications of ERα, which include acetylation, methylation, ubiquitination and SUMOylation (Ascenzi *et al*. 2006, Le Romancer *et al*. 2011), may also affect the sensitivity of breast cancer cells to AEs.

### Induction of ERα ubiquitination by SERDs

SERDs (ICI 182,780, RU 58,668 and GW7604) accelerate ERα degradation in uterine and breast cancer cell lines. Degradation takes place with faster kinetics than that in the presence of agonists in MCF-7 cells. Although 4-hydroxytamoxifen stabilizes ERα protein levels (Wijayaratne & McDonnell 2001, Lupien *et al*. 2007, Kocanova *et al*. 2010, Hilmi *et al*. 2012), decreased steady-state levels of ERα were observed to variable extents in the presence of endoxifen (a tamoxifen metabolite), raloxifene and bazedoxifene. However, none of these AEs were as efficacious as the pure AE ICI 182,780 (Wardell *et al*. 2013). Affinity purification of ERα modified by tagged ubiquitin showed that ICI 182,780 triggers a 2-fold enhancement of ERα ubiquitination compared with basal levels (Wijayaratne & McDonnell 2001). Agonists induce or are permissive for recruitment of several E3 ubiquitin ligases, such as E6-AP, CHIP, MDM2, BRCA1/BARD1, EFP/TRIM25, SPOP, RBCK1, CUEDC2, SKP2, VHL and RNF31 by ERα (Helzer *et al*. 2015 and refs within); some of these proteins are recruited to DNA and can act as ERα coactivators (Lonard *et al*. 2000, Reid *et al*. 2003). However, E3 ligases recruited in the presence of SERDs still need to be characterized.

Mechanisms of degradation appear to differ in the presence of AEs and estradiol. Inhibition of transcription by α-amanitin or other inhibitors prevents the induction of ERα turnover by agonists but not by ICI 182,780 (Reid *et al*. 2003). In addition, cycloheximide treatment and several kinase inhibitors (PKA, PI3K) partially blocked the induction of ERα protein turnover by estradiol but not pure AEs (Borras *et al*. 1994, 1996, Seo *et al*. 1998, Marsaud *et al*. 2003). Furthermore, overexpression of ERα in breast cancer cells can saturate the degradation process in the presence of SERDs, without affecting turnover in the presence of agonists (Wardell *et al*. 2011). Finally, removal or mutation of the nuclear localization signal (NLS) in ERα, resulting in cytoplasmic localization of the receptor, abolished degradation in the presence of ICI 182,780 but not estradiol, while adding back the endogenous NLS to the N-terminus of ERα partially restored the degradation of ERα in the presence of ICI 182,780 (Casa *et al*. 2015). In spite of the above-mentioned differences between degradation mechanisms in the presence of estradiol and SERDs, the Neddylation pathway, which resembles the ubiquitination cascade and cooperates with it for induction of ubiquitination, appears to be important for both estradiol- and ICI 182,780-induced degradation (Fan *et al*. 2003). Mapping of residues affected by polyubiquitination in the presence of SERDs and identification of E3 ligases and deubiquitinases controlling receptor modification by the ubiquitin system should clarify the similitudes and differences between the mechanisms of receptor degradation in the presence of SERDs vs agonists.

### Induction of ERα SUMOylation by SERDs

Although induction of ERα degradation is expected to contribute to pure antiestrogenicity, saturating the degradation process by overexpressing ERα did not appear to affect the capacity of SERDs (bazedoxifene, ICI 182,780 and GW7604) to act as AEs in MCF-7 cells (Wardell *et al*. 2011). Further, although the steady-state levels of transfected ERα were not decreased but rather increased in the presence of ICI 182,780 in HepG2 cells, ICI 182,780 still acted as an inverse agonist in these cells, whereas tamoxifen had partial agonist activity (Lupien *et al*. 2007, Hilmi *et al*. 2012). This suggests the existence of other mechanisms for the increased efficacy of pure AEs in inhibiting ERα activity in these cells. In this respect, we have observed that pure AEs strongly induce ERα SUMOylation in the MCF-7 breast cancer cell line, as well as in transiently transfected HEK293 and HepG2 cells (Hilmi *et al*. 2012). Abrogating SUMOylation by overexpression of the SENP1 deSUMOylase partially derepressed transcription in the presence of pure AEs in HepG2 cells without an increase in the corresponding activity with estradiol or tamoxifen, suggesting that induction of ERα SUMOylation contributes to pure antiestrogenicity (Hilmi *et al*. 2012).

Interestingly, SUMOylation correlated with pure antiestrogenicity in a panel of molecules derived from ICI 164,384. SUMOylation activity was observed with chains harboring 14 carbon atoms, reached maximal levels with chain lengths between 15 and 19 and then diminished with chain lengths of 22 atoms, correlating with inverse agonist activity in HepG2 cells and with the capacity of the AE side chain to interact with the coactivator-binding groove in molecular models (Fig. 4 and Video 1). In addition to pure AEs, the SERM raloxifene was shown to induce SUMOylation to a lower degree, correlating with its capacity to suppress basal transcriptional activity in HepG2 cells (Hilmi *et al*. 2012). Thus, differential SUMOylation may also contribute to the differential SERM profiles in different tissues.

SUMOylation may affect cofactor recruitment by ERα. Indeed, SUMOylated androgen and glucocorticoid receptors can bind the corepressor DAXX, which in turn recruits chromatin-modifying enzymes (HDACs) or DNA methyltransferases to inhibit transcriptional activity of nuclear receptors (Shih *et al*. 2007). Another function of SUMOylation is its capacity to recruit SUMO-targeted ubiquitin ligases (STUbLs), such as RNF4 in humans, to promote the degradation of the modified protein, as shown for PML (Heideker *et al*. 2009). There could therefore be a link between SUMOylation of ERα and its increased degradation rate in the presence of SERDs. Indeed, the low level of ERα SUMOylation in the presence of raloxifene (Hilmi *et al*. 2012) correlates with the weak induction of degradation by this AE. Studying the modification pattern of ERα in the presence of bazedoxifene and GW7604 would further help assess this hypothesis.

Mass spectrometric analyses led to the identification of four SUMOylation sites in ERα in the presence of ICI 182,780: Lys171 and Lys180 located just upstream of the DBD, Lys299 in the hinge region and Lys472 in the LBD (Fig. 2A); however, combined mutagenesis of these sites did not abolish the SUMOylation of ERα in the presence of ICI 182,780 (Hilmi *et al*. 2012), suggesting that other sites remain to be discovered. Characterization of mutants that inhibit SUMOylation will be important to further investigate the link between SUMOylation and ubiquitination, as well as the role of each type of modification in pure antiestrogenicity.

## Impact of ERα mutations found in endocrine treatment-resistant tumors on AE action

Development of resistance to endocrine treatment is a major outstanding issue for ER+ breast cancer patients. About 25% of ER+ patients with early-stage disease will develop resistance to endocrine treatment within 10 years of diagnosis (EBCTCG 2005), and all metastatic patients will eventually progress on endocrine treatment. Notably, expression of ERα is preserved in the majority of tumors after development of resistance (Johnston 1997), suggesting a continued role of ERα in tumor progression. Overexpression of coactivators driving estrogen-dependent transcription is a potential mechanism for this loss of sensitivity, as is activation of signaling pathways that modulate the activity of ERα and/or its coactivators (Johnston 1997, Nardone *et al*. 2015). Recently, ERα mutations have emerged as an additional mechanism of resistance to hormonal treatment (see Jeselsohn *et al*. 2015 for a review). This was first suggested by the isolation of a constitutively active ERα mutant (Y537N) from a breast metastasis (Zhang *et al*. 1997). More recently, several studies have reported the identification of mutations in the ERα LBD in metastases of patients having undergone at least one line of endocrine treatment (Li *et al*. 2013, Merenbakh-Lamin *et al*. 2013, Robinson *et al*. 2013, Toy *et al*. 2013, Jeselsohn *et al*. 2014). Importantly, these mutations can be detected by isolation of circulating tumor DNA in the blood of breast cancer patients (Guttery *et al*. 2015, Sefrioui *et al*. 2015) and may serve to orient therapeutic decision.

Most mutations characterized in tumors resistant to endocrine therapies are gain-of-function mutations (e.g. E380Q, L536Q/R, D538G and Y537S/C/N) that result in ligand-independent ERα activity in reporter gene assays or on endogenous estrogen target genes (e.g. *GREB1*, *PGR*, *TFF1*, *MYC* and *XBP1*) (Li *et al*. 2013, Robinson *et al*. 2013, Toy *et al*. 2013, Jeselsohn *et al*. 2014). Of interest, several of these mutations or additional ones at the same positions had been previously characterized as leading to increased basal activity in functional analyses of ER signaling (Pakdel *et al*. 1993, Weis *et al*. 1996, Eng *et al*. 1997). Constitutive mutants demonstrate increased levels of Ser118 phosphorylation, resistance to HSP90 inhibitor-induced degradation, enhanced recruitment of NCOA family coactivators and/or increased ligand-independent tumor growth in xenograft models compared with wt ERα (Merenbakh-Lamin *et al*. 2013, Toy *et al*. 2013, Fanning *et al*. 2016). Ligand-independent growth of tumors was also seen in patient-derived xenografts (PDX) established from metastatic ER+ tumors harboring the Y537S mutation (Li *et al*. 2013).

Y537S- and D538G-mutant ERα LBDs adopt an agonist-like conformation in the absence of ligand in molecular models and in crystal structures (Nettles *et al*. 2008, Merenbakh-Lamin *et al*. 2013, Toy *et al*. 2013, Fanning *et al*. 2016). As H12 acts as a lid to the ligand-binding cavity in the agonist conformation, its stabilization in this position in the unliganded ERα due to mutations should affect binding of ER ligands including AEs. Indeed, affinity of mutants Y537S and D538G for estradiol and 4-hydroxytamoxifen was 5- to 10-fold smaller than for wt ERα (Fanning *et al*. 2016). Accordingly, higher doses of 4-hydroxytamoxifen and ICI 182,780 were required to inhibit the activity of mutant ERα to levels comparable with those observed with the wt ERα; this may lead to resistance to treatment with AEs in the clinic if concentrations high enough to suppress activity of the mutants cannot be achieved (Merenbakh-Lamin *et al*. 2013, Toy *et al*. 2013, Jeselsohn *et al*. 2014). In addition, the altered structures of the mutant ERα LBDs in the presence of 4-hydroxytamoxifen (Fanning *et al*. 2016) may lead to different impacts on ER target genes at saturation than with the wt receptor. Finally, it is worth noting that mutation L536A, but not Y537A, was found to increase ERα transcriptional activity and to decrease receptor SUMOylation in the presence of ICI 182,780 (Lupien *et al*. 2007, and our unpublished data). It will therefore be of interest in the future to determine to which extent each of the ERα LBD mutations associated with resistance to endocrine therapies affects the efficacy of pure AEs in suppressing ER target gene expression to better guide the choice of second-line therapies.

## Conclusion

Structural and functional studies have revealed that AEs use a diversity of conformational solutions to modulate AF2 and/or AF1 activity. This results in varying degrees of antiestrogenicity in breast cancer cells, and in different patterns of tissue-specific activity. How each conformation or change in conformational dynamics is linked to functional effects such as alterations in receptor ubiquitination and SUMOylation, recruitment of specific cofactors, release from DNA and degradation still remains to be further explored. Ultimately, the relevance of these questions to the clinic will be informed by the characterization of orally bioavailable SERDs in both second- and first-line treatment of breast cancer. In addition, recombinant cell lines and PDX models of endocrine therapy resistance due to ERα mutations should prove extremely useful to better characterize the response patterns of each of these ERα mutants to existing AEs and to develop novel, more effective therapeutic molecules or drug combinations.

## Declaration of interest

The authors declare that there is no conflict of interest that could be perceived as prejudicing the impartiality of this review.

## Funding

This work has been funded by the Canadian Institute for Health Research (grant 125863 to SM) and from the Canadian Imperial Bank of Commerce (CIBC) breast cancer research chair at Université de Montréal (to SM).

## References

[bib1] Early Breast Cancer Trialists’ Collaborative Group (EBCTCG) 2005 Effects of chemotherapy and hormonal therapy for early breast cancer on recurrence and 15-year survival: an overview of the randomised trials. Lancet 365 1687–1717. (10.1016/S0140-6736(05)66544-0) 15894097

[bib2] Ali S Buluwela L Coombes RC 2011 Antiestrogens and their therapeutic applications in breast cancer and other diseases. Annual Review of Medicine 62 217–232. (10.1146/annurev-med-052209-100305) 21054173

[bib3] Ali S Metzger D Bornert J-M Chambon P 1993 Modulation of transcriptional activation by ligand-dependent phosphorylation of the estrogen receptor A/B region. EMBO Journal 12 1153–1160. 8458328 10.1002/j.1460-2075.1993.tb05756.x PMC413317

[bib4] Aranda A Pascual A 2001 Nuclear hormone receptors and gene expression. Physiological Reviews 81 1269–1304. 11427696 10.1152/physrev.2001.81.3.1269

[bib5] Arao Y Coons LA Zuercher WJ Korach KS 2015 Transactivation function-2 of estrogen receptor alpha contains transactivation function-1-regulating element. Journal of Biological Chemistry 290 17611–17627. (10.1074/jbc.M115.638650) 26028650 PMC4498094

[bib6] Arao Y Hamilton KJ Ray MK Scott G Mishina Y Korach KS 2011 Estrogen receptor alpha AF-2 mutation results in antagonist reversal and reveals tissue selective function of estrogen receptor modulators. PNAS 108 14986–14991. (10.1073/pnas.1109180108) 21873215 PMC3169108

[bib7] Ascenzi P Bocedi A Marino M 2006 Structure–function relationship of estrogen receptor alpha and beta: impact on human health. Molecular Aspects of Medicine 27 299–402. (10.1016/j.mam.2006.07.001) 16914190

[bib8] Barsalou A Gao W Anghel S Carriere J Mader S 1998 Estrogen response elements can mediate agonist activity of antiestrogens in human endometrial Ishikawa cells. Journal of Biological Chemistry 273 17138–17146. (10.1074/jbc.273.27.17138) 9642281

[bib9] Benecke A Chambon P Gronemeyer H 2000 Synergy between estrogen receptor alpha activation functions AF1 and AF2 mediated by transcription intermediary factor TIF2. EMBO Reports 1 151–157. (10.1093/embo-reports/kvd028) 11265755 PMC1084260

[bib10] Bentrem D Dardes R Liu H MacGregor-Schafer J Zapf J Jordan V 2001 Molecular mechanism of action at estrogen receptor alpha of a new clinically relevant antiestrogen (GW7604) related to tamoxifen. Endocrinology 142 838–846. (10.1210/en.142.2.838) 11159857

[bib11] Berry M Metzger D Chambon P 1990 Role of the two activating domains of the oestrogen receptor in the cell-type and promoter-context dependent agonistic activity of the anti-oestrogen 4-hydroxytamoxifen. EMBO Journal 9 2811–2818. 2118104 10.1002/j.1460-2075.1990.tb07469.x PMC551992

[bib12] Black LJ Sato M Rowley ER Magee DE Bekele A Williams DC Cullinan GJ Bendele R Kauffman RF Bensch WR 1994 Raloxifene (LY139481 HCI) prevents bone loss and reduces serum cholesterol without causing uterine hypertrophy in ovariectomized rats. Journal of Clinical Investigation 93 63–69. (10.1172/JCI116985) 8282823 PMC293730

[bib13] Borras M Hardy L Lempereur F el Khissiin AH Legros N Gol-Winkler R Leclercq G 1994 Estradiol-induced down-regulation of estrogen receptor. Effect of various modulators of protein synthesis and expression. Journal of Steroid Biochemistry and Molecular Biology 48 325–336. (10.1016/0960-0760(94)90072-8) 8142311

[bib14] Borras M Laios I el Khissiin A Seo HS Lempereur F Legros N Leclercq G 1996 Estrogenic and antiestrogenic regulation of the half-life of covalently labeled estrogen receptor in MCF-7 breast cancer cells. Journal of Steroid Biochemistry and Molecular Biology 57 203–213. (10.1016/0960-0760(95)00272-3) 8645630

[bib15] Bowler J Lilley TJ Pittam JD Wakeling AE 1989 Novel steroidal pure antiestrogens. Steroids 54 71–99. (10.1016/0039-128X(89)90076-7) 2815158

[bib16] Brzozowski AM Pike AC Dauter Z Hubbard RE Bonn T Engstrom O Ohman L Greene GL Gustafsson JA Carlquist M 1997 Molecular basis of agonism and antagonism in the oestrogen receptor. Nature 389 753–758. (10.1038/39645) 9338790

[bib17] Burandt E Jens G Holst F Janicke F Muller V Quaas A Choschzick M Wilczak W Terracciano L Simon R 2013 Prognostic relevance of AIB1 (NCoA3) amplification and overexpression in breast cancer. Breast Cancer Research and Treatment 137 745–753. (10.1007/s10549-013-2406-4) 23322234

[bib18] Callis R Rabow A Tonge M Bradbury R Challinor M Roberts K Jones K Walker G 2015 A screening assay cascade to identify and characterize novel selective estrogen receptor downregulators (SERDs). Journal of Biomolecular Screening 20 748–759. (10.1177/1087057115580298) 25851036

[bib19] Casa AJ Hochbaum D Sreekumar S Oesterreich S Lee AV 2015 The estrogen receptor alpha nuclear localization sequence is critical for fulvestrant-induced degradation of the receptor. Molecular and Cellular Endocrinology 415 76–86. (10.1016/j.mce.2015.08.007) 26272024

[bib20] Chang KC Wang Y Bodine PV Nagpal S Komm BS 2010 Gene expression profiling studies of three SERMs and their conjugated estrogen combinations in human breast cancer cells: insights into the unique antagonistic effects of bazedoxifene on conjugated estrogens. Journal of Steroid Biochemistry and Molecular Biology 118 117–124. (10.1016/j.jsbmb.2009.11.003) 19914376

[bib21] Ciana P Di Luccio G Belcredito S Pollio G Vegeto E Tatangelo L Tiveron C Maggi A 2001 Engineering of a mouse for the in vivo profiling of estrogen receptor activity. Molecular Endocrinology 15 1104–1113. (10.1210/mend.15.7.0658) 11435611

[bib22] Cirillo F Nassa G Tarallo R Stellato C De Filippo MR Ambrosino C Baumann M Nyman TA Weisz A 2013 Molecular mechanisms of selective estrogen receptor modulator activity in human breast cancer cells: identification of novel nuclear cofactors of antiestrogen-ERalpha complexes by interaction proteomics. Journal of Proteome Research 12 421–431. (10.1021/pr300753u) 23170835

[bib23] Dauvois S Danielian PS White R Parker MG 1992 Antiestrogen ICI 164,384 reduces cellular estrogen receptor content by increasing its turnover. PNAS 89 4037–4041. (10.1073/pnas.89.9.4037) 1570330 PMC525627

[bib24] Davies P Syne JS Nicholson RI 1979 Effects of estradiol and the antiestrogen tamoxifen on steroid hormone receptor concentration and nuclear ribonucleic acid polymerase activities in rat uteri. Endocrinology 105 1336–1342. (10.1210/endo-105-6-1336) 499077

[bib25] Dayan G Lupien M Auger A Anghel SI Rocha W Croisetiere S Katzenellenbogen JA Mader S 2006 Tamoxifen and raloxifene differ in their functional interactions with aspartate 351 of estrogen receptor alpha. Molecular Pharmacology 70 579–588. (10.1124/mol.105.021931) 16679488

[bib26] Deroo BJ Korach KS 2006 Estrogen receptors and human disease. Journal of Clinical Investigation 116 561–570. (10.1172/JCI27987) 16511588 PMC2373424

[bib27] Deshmane V Krishnamurthy S Melemed AS Peterson P Buzdar AU 2007 Phase III double-blind trial of arzoxifene compared with tamoxifen for locally advanced or metastatic breast cancer. Journal of Clinical Oncology 25 4967–4973. (10.1200/JCO.2006.09.5992) 17971595

[bib28] Di Leo A Jerusalem G Petruzelka L Torres R Bondarenko IN Khasanov R Verhoeven D Pedrini JL Smirnova I Lichinitser MR 2010 Results of the CONFIRM phase III trial comparing fulvestrant 250 mg with fulvestrant 500 mg in postmenopausal women with estrogen receptor-positive advanced breast cancer. Journal of Clinical Oncology 28 4594–4600. (10.1200/JCO.2010.28.8415) 20855825

[bib29] Di Leo A Jerusalem G Petruzelka L Torres R Bondarenko IN Khasanov R Verhoeven D Pedrini JL Smirnova I Lichinitser MR 2014 Final overall survival: fulvestrant 500 mg vs 250 mg in the randomized CONFIRM trial. Journal of the National Cancer Institute 106 djt337 (10.1093/jnci/djt337) 24317176 PMC3906991

[bib30] El Khissiin A Leclercq G 1999 Implication of proteasome in estrogen receptor degradation. FEBS Letters 448 160–166. (10.1016/S0014-5793(99)00343-9) 10217432

[bib31] Eng FC Lee HS Ferrara J Willson TM White JH 1997 Probing the structure and function of the estrogen receptor ligand binding domain by analysis of mutants with altered transactivation characteristics. Molecular and Cellular Biology 17 4644–4653. (10.1128/MCB.17.8.4644) 9234721 PMC232317

[bib32] Fan M Bigsby RM Nephew KP 2003 The NEDD8 pathway is required for proteasome-mediated degradation of human estrogen receptor (ER)-alpha and essential for the antiproliferative activity of ICI 182,780 in ERalpha-positive breast cancer cells. Molecular Endocrinology 17 356–365. (10.1210/me.2002-0323) 12554766

[bib33] Fanning SW Mayne CG Dharmarajan V Carlson KE Martin TA Novick SJ Toy W Green B Panchamukhi S Katzenellenbogen BS 2016 Estrogen receptor alpha somatic mutations Y537S and D538G confer breast cancer endocrine resistance by stabilizing the activating function-2 binding conformation. eLife 5 e12792. (10.7554/elife.12792) 26836308 PMC4821807

[bib34] Frasor J Stossi F Danes JM Komm B Lyttle CR Katzenellenbogen BS 2004 Selective estrogen receptor modulators: discrimination of agonistic versus antagonistic activities by gene expression profiling in breast cancer cells. Cancer Research 64 1522–1533. (10.1158/0008-5472.CAN-03-3326) 14973112

[bib35] Gallagher A Chambers TJ Tobias JH 1993 The estrogen antagonist ICI 182,780 reduces cancellous bone volume in female rats. Endocrinology 133 2787–2791. (10.1210/en.133.6.2787) 8243306

[bib36] Gibson MK Nemmers LA Beckman WC Jr Davis VL Curtis SW Korach KS 1991 The mechanism of ICI 164,384 antiestrogenicity involves rapid loss of estrogen receptor in uterine tissue. Endocrinology 129 2000–2010. (10.1210/endo-129-4-2000) 1915080

[bib37] Grese TA Cho S Finley DR Godfrey AG Jones CD Lugar CW 3rd Martin MJ Matsumoto K Pennington LD Winter MA 1997 Structure–activity relationships of selective estrogen receptor modulators: modifications to the 2-arylbenzothiophene core of raloxifene. Journal of Medicinal Chemistry 40 146–167. (10.1021/jm9606352) 9003514

[bib38] Guttery DS Page K Hills A Woodley L Marchese SD Rghebi B Hastings RK Luo J Pringle JH Stebbing J 2015 Noninvasive detection of activating estrogen receptor 1 (ESR1) mutations in estrogen receptor-positive metastatic breast cancer. Clinical Chemistry 61 974–982. (10.1373/clinchem.2015.238717) 25979954

[bib39] Hall JM Couse JF Korach KS 2001 The multifaceted mechanisms of estradiol and estrogen receptor signaling. Journal of Biological Chemistry 276 36869–36872. (10.1074/jbc.R100029200) 11459850

[bib40] Hall JM McDonnell DP 2005 Coregulators in nuclear estrogen receptor action: from concept to therapeutic targeting. Molecular Interventions 5 343–357. (10.1124/mi.5.6.7) 16394250

[bib41] Heideker J Perry JJ Boddy MN 2009 Genome stability roles of SUMO-targeted ubiquitin ligases. DNA Repair 8 517–524. (10.1016/j.dnarep.2009.01.010) 19233742 PMC2685196

[bib42] Heldring N Pawson T McDonnell D Treuter E Gustafsson JA Pike AC 2007 Structural insights into corepressor recognition by antagonist-bound estrogen receptors. Journal of Biological Chemistry 282 10449–10455. (10.1074/jbc.M611424200) 17283072

[bib43] Helzer KT Hooper C Miyamoto S Alarid ET 2015 Ubiquitylation of nuclear receptors: new linkages and therapeutic implications. Journal of Molecular Endocrinology 54 R151–R167. (10.1530/JME-14-0308) 25943391 PMC4457637

[bib44] Hilmi K Hussein N Mendoza-Sanchez R El-Ezzy M Ismail H Durette C Bail M Rozendaal MJ Bouvier M Thibault P 2012 Role of SUMOylation in full antiestrogenicity. Molecular and Cellular Biology 32 3823–3837. (10.1128/MCB.00290-12) 22826433 PMC3457522

[bib45] Hoffman KL Foster EA Smith CL 2012 The terminal substituents of 7alpha, 6-hexanyl derivatives of estradiol determine their selective estrogen receptor modulator versus agonist activities. Steroids 77 496–503. (10.1016/j.steroids.2012.01.011) 22326682 PMC3303951

[bib46] Hoffmann J Bohlmann R Heinrich N Hofmeister H Kroll J Kunzer H Lichtner RB Nishino Y Parczyk K Sauer G 2004 Characterization of new estrogen receptor destabilizing compounds: effects on estrogen-sensitive and tamoxifen-resistant breast cancer. Journal of the National Cancer Institute 96 210–218. (10.1093/jnci/djh022) 14759988

[bib47] Howell A 2006 Pure oestrogen antagonists for the treatment of advanced breast cancer. Endocrine-Related Cancer 13 689–706. (10.1677/erc.1.00846) 16954425

[bib48] Howell A Robertson JF Abram P Lichinitser MR Elledge R Bajetta E Watanabe T Morris C Webster A Dimery I 2004a Comparison of fulvestrant versus tamoxifen for the treatment of advanced breast cancer in postmenopausal women previously untreated with endocrine therapy: a multinational, double-blind, randomized trial. Journal of Clinical Oncology 22 1605–1613. (10.1200/JCO.2004.02.112) 15117982

[bib49] Howell SJ Johnston SR Howell A 2004b The use of selective estrogen receptor modulators and selective estrogen receptor down-regulators in breast cancer. Best Practice and Research: Clinical Endocrinology and Metabolism 18 47–66. (10.1016/j.beem.2003.08.002) 14687597

[bib50] Howell A Robertson JF Quaresma Albano J Aschermannova A Mauriac L Kleeberg UR Vergote I Erikstein B Webster A Morris C 2002 Fulvestrant, formerly ICI 182,780, is as effective as anastrozole in postmenopausal women with advanced breast cancer progressing after prior endocrine treatment. Journal of Clinical Oncology 20 3396–3403. (10.1200/JCO.2002.10.057) 12177099

[bib51] Hu XF Veroni M De Luise M Wakeling A Sutherland R Watts CK Zalcberg JR 1993 Circumvention of tamoxifen resistance by the pure anti-estrogen ICI 182,780. International Journal of Cancer 55 873–876. (10.1002/ijc.2910550529) 8244585

[bib52] Janknecht R 2010 Multi-talented DEAD-box proteins and potential tumor promoters: p68 RNA helicase (DDX5) and its paralog, p72 RNA helicase (DDX17). American Journal of Translational Research 2 223–234. 20589163 PMC2892403

[bib53] Jeselsohn R Buchwalter G De Angelis C Brown M Schiff R 2015 ESR1 mutations-a mechanism for acquired endocrine resistance in breast cancer. Nature Reviews Clinical Oncology 12 573–583. (10.1038/nrclinonc.2015.117) PMC4911210 26122181

[bib54] Jeselsohn R Yelensky R Buchwalter G Frampton G Meric-Bernstam F Gonzalez-Angulo AM Ferrer-Lozano J Perez-Fidalgo JA Cristofanilli M Gomez H 2014 Emergence of constitutively active estrogen receptor-alpha mutations in pretreated advanced estrogen receptor-positive breast cancer. Clinical Cancer Research 20 1757–1767. (10.1158/1078-0432.CCR-13-2332) 24398047 PMC3998833

[bib55] Jia M Dahlman-Wright K Gustafsson JA 2015 Estrogen receptor alpha and beta in health and disease. Best Practice and Research: Clinical Endocrinology and Metabolism 29 557–568. (10.1016/j.beem.2015.04.008) 26303083

[bib56] Johnson AB O’Malley BW 2012 Steroid receptor coactivators 1, 2, and 3: critical regulators of nuclear receptor activity and steroid receptor modulator (SRM)-based cancer therapy. Molecular and Cellular Endocrinology 348 430–439. (10.1016/j.mce.2011.04.021) 21664237 PMC3202666

[bib57] Johnston SR 1997 Acquired tamoxifen resistance in human breast cancer – potential mechanisms and clinical implications. Anticancer Drugs 8 911–930. (10.1097/00001813-199711000-00002) 9436634

[bib58] Jordan VC 2004 Selective estrogen receptor modulation: concept and consequences in cancer. Cancer Cell 5 207–213. (10.1016/S1535-6108(04)00059-5) 15050912

[bib59] Keeton EK Brown M 2005 Cell cycle progression stimulated by tamoxifen-bound estrogen receptor-alpha and promoter-specific effects in breast cancer cells deficient in N-CoR and SMRT. Molecular Endocrinology 19 1543–1554. (10.1210/me.2004-0395) 15802375

[bib60] Klotz DM Hewitt SC Korach KS Diaugustine RP 2000 Activation of a uterine insulin-like growth factor I signaling pathway by clinical and environmental estrogens: requirement of estrogen receptor-alpha. Endocrinology 141 3430–3439. (10.1210/en.141.9.3430) 10965916

[bib61] Kocanova S Mazaheri M Caze-Subra S Bystricky K 2010 Ligands specify estrogen receptor alpha nuclear localization and degradation. BMC Cell Biology 11 98 (10.1186/1471-2121-11-98) 21143970 PMC3009626

[bib62] Korach KS 1994 Insights from the study of animals lacking functional estrogen receptor. Science 266 1524–1527. (10.1126/science.7985022) 7985022

[bib63] Lai A Kahraman M Govek S Nagasawa J Bonnefous C Julien J Douglas K Sensintaffar J Lu N Lee KJ 2015 Identification of GDC-0810 (ARN-810), an orally bioavailable selective estrogen receptor degrader (SERD) that demonstrates robust activity in tamoxifen-resistant breast cancer xenografts. Journal of Medicinal Chemistry 58 4888–4904. (10.1021/acs.jmedchem.5b00054) 25879485

[bib64] Lanz RB McKenna NJ Onate SA Albrecht U Wong J Tsai SY Tsai MJ O’Malley BW 1999 A steroid receptor coactivator, SRA, functions as an RNA and is present in an SRC-1 complex. Cell 97 17–27. (10.1016/S0092-8674(00)80711-4) 10199399

[bib65] Lavinsky RM Jepsen K Heinzel T Torchia J Mullen TM Schiff R Del-Rio AL Ricote M Ngo S Gemsch J 1998 Diverse signaling pathways modulate nuclear receptor recruitment of N-CoR and SMRT complexes. PNAS 95 2920–2925. (10.1073/pnas.95.6.2920) 9501191 PMC19670

[bib66] Le Romancer M Poulard C Cohen P Sentis S Renoir JM Corbo L 2011 Cracking the estrogen receptor’s posttranslational code in breast tumors. Endocrine Reviews 32 597–622. (10.1210/er.2010-0016) 21680538

[bib67] Li S Shen D Shao J Crowder R Liu W Prat A He X Liu S Hoog J Lu C 2013 Endocrine-therapy-resistant ESR1 variants revealed by genomic characterization of breast-cancer-derived xenografts. Cell Reports 4 1116–1130. (10.1016/j.celrep.2013.08.022) 24055055 PMC3881975

[bib68] Liu XF Bagchi MK 2004 Recruitment of distinct chromatin-modifying complexes by tamoxifen-complexed estrogen receptor at natural target gene promoters in vivo. Journal of Biological Chemistry 279 15050–15058. (10.1074/jbc.M311932200) 14722073

[bib69] Liu H Park WC Bentrem DJ McKian KP Reyes Ade L Loweth JA Schafer JM Zapf JW Jordan VC 2002 Structure–function relationships of the raloxifene-estrogen receptor-alpha complex for regulating transforming growth factor-alpha expression in breast cancer cells. Journal of Biological Chemistry 277 9189–9198. (10.1074/jbc.M108335200) 11751902 PMC3696956

[bib70] Lonard DM Nawaz Z Smith CL O’Malley BW 2000 The 26S proteasome is required for estrogen receptor-alpha and coactivator turnover and for efficient estrogen receptor-alpha transactivation. Molecular Cell 5 939–948. (10.1016/S1097-2765(00)80259-2) 10911988

[bib71] Long X Fan M Nephew KP 2010 Estrogen receptor-alpha-interacting cytokeratins potentiate the antiestrogenic activity of fulvestrant. Cancer Biology and Therapy 9 389–396. (10.4161/cbt.9.5.10926) 20061804

[bib72] Long X Nephew KP 2006 Fulvestrant (ICI 182,780)-dependent interacting proteins mediate immobilization and degradation of estrogen receptor-alpha. Journal of Biological Chemistry 281 9607–9615. (10.1074/jbc.M510809200) 16459337

[bib73] Louie MC Zou JX Rabinovich A Chen HW 2004 ACTR/AIB1 functions as an E2F1 coactivator to promote breast cancer cell proliferation and antiestrogen resistance. Molecular and Cellular Biology 24 5157–5171. (10.1128/MCB.24.12.5157-5171.2004) 15169882 PMC419858

[bib74] Love RR Barden HS Mazess RB Epstein S Chappell RJ 1994a Effect of tamoxifen on lumbar spine bone mineral density in postmenopausal women after 5 years. Archives of Internal Medicine 154 2585–2588. (10.1001/archinte.1994.00420220081009) 7979855

[bib75] Love RR Wiebe DA Feyzi JM Newcomb PA Chappell RJ 1994b Effects of tamoxifen on cardiovascular risk factors in postmenopausal women after 5 years of treatment. Journal of the National Cancer Institute 86 1534–1539. (10.1093/jnci/86.20.1534) 7932809

[bib76] Lupien M Jeyakumar M Hebert E Hilmi K Cotnoir-White D Loch C Auger A Dayan G Pinard GA Wurtz JM 2007 Raloxifene and ICI182,780 increase estrogen receptor-alpha association with a nuclear compartment via overlapping sets of hydrophobic amino acids in activation function 2 helix 12. Molecular Endocrinology 21 797–816. (10.1210/me.2006-0074) 17299137

[bib77] Lykkesfeldt AE Larsen SS Briand P 1995 Human breast cancer cell lines resistant to pure anti-estrogens are sensitive to tamoxifen treatment. International Journal of Cancer 61 529–534. (10.1002/ijc.2910610417) 7759159

[bib78] Lykkesfeldt AE Madsen MW Briand P 1994 Altered expression of estrogen-regulated genes in a tamoxifen-resistant and ICI 164,384 and ICI 182,780 sensitive human breast cancer cell line, MCF-7/TAMR-1. Cancer Research 54 1587–1595. 8137264

[bib79] MacGregor Schafer J Liu H Bentrem DJ Zapf JW Jordan VC 2000 Allosteric silencing of activating function 1 in the 4-hydroxytamoxifen estrogen receptor complex is induced by substituting glycine for aspartate at amino acid 351. Cancer Research 60 5097–5105. 11016635

[bib80] Mahfoudi A Roulet E Dauvois S Parker MG Wahli W 1995 Specific mutations in the estrogen receptor change the properties of antiestrogens to full agonists. PNAS 92 4206–4210. (10.1073/pnas.92.10.4206) 7753783 PMC41912

[bib81] Marsaud V Gougelet A Maillard S Renoir JM 2003 Various phosphorylation pathways, depending on agonist and antagonist binding to endogenous estrogen receptor alpha (ERalpha), differentially affect ERalpha extractability, proteasome-mediated stability, and transcriptional activity in human breast cancer cells. Molecular Endocrinology 17 2013–2027. (10.1210/me.2002-0269) 12855746

[bib82] Martin L Middleton E 1978 Prolonged oestrogenic and mitogenic activity of tamoxifen in the ovariectomized mouse. Journal of Endocrinology 78 125–129. (10.1677/joe.0.0780125) 681866

[bib83] Martinkovich S Shah D Planey SL Arnott JA 2014 Selective estrogen receptor modulators: tissue specificity and clinical utility. Clinical Interventions in Aging 9 1437–1452. (10.2147/CIA.S66690) 25210448 PMC4154886

[bib84] McDonnell DP Wardell SE Norris JD 2015 Oral selective estrogen receptor downregulators (SERDs), a breakthrough endocrine therapy for breast cancer. Journal of Medicinal Chemistry 58 4883–4887. (10.1021/acs.jmedchem.5b00760) 26039356 PMC4708081

[bib85] McInerney EM Weis KE Sun J Mosselman S Katzenellenbogen BS 1998 Transcription activation by the human estrogen receptor subtype beta (ER beta) studied with ER beta and ER alpha receptor chimeras. Endocrinology 139 4513–4522. (10.1210/en.139.11.4513) 9794460

[bib86] Merenbakh-Lamin K Ben-Baruch N Yeheskel A Dvir A Soussan-Gutman L Jeselsohn R Yelensky R Brown M Miller VA Sarid D 2013 D538G mutation in estrogen receptor-alpha: a novel mechanism for acquired endocrine resistance in breast cancer. Cancer Research 73 6856–6864. (10.1158/0008-5472.CAN-13-1197) 24217577

[bib87] Merot Y Metivier R Penot G Manu D Saligaut C Gannon F Pakdel F Kah O Flouriot G 2004 The relative contribution exerted by AF-1 and AF-2 transactivation functions in estrogen receptor alpha transcriptional activity depends upon the differentiation stage of the cell. Journal of Biological Chemistry 279 26184–26191. (10.1074/jbc.M402148200) 15078875

[bib88] Metivier R Penot G Flouriot G Pakdel F 2001 Synergism between ERalpha transactivation function 1 (AF-1) and AF-2 mediated by steroid receptor coactivator protein-1: requirement for the AF-1 alpha-helical core and for a direct interaction between the N- and C-terminal domains. Molecular Endocrinology 15 1953–1970. (10.1210/me.15.11.1953) 11682626

[bib89] Miller PD Chines AA Christiansen C Hoeck HC Kendler DL Lewiecki EM Woodson G Levine AB Constantine G Delmas PD 2008 Effects of bazedoxifene on BMD and bone turnover in postmenopausal women: 2-year results of a randomized, double-blind, placebo-, and active-controlled study. Journal of Bone and Mineral Research 23 525–535. (10.1359/jbmr.071206) 18072873

[bib90] Moras D Gronemeyer H 1998 The nuclear receptor ligand-binding domain: structure and function. Current Opinion in Cell Biology 10 384–391. (10.1016/S0955-0674(98)80015-X) 9640540

[bib91] Musgrove EA Sutherland RL 2009 Biological determinants of endocrine resistance in breast cancer. Nature Reviews Cancer 9 631–643. (10.1038/nrc2713) 19701242

[bib92] Nardone A De Angelis C Trivedi MV Osborne CK Schiff R 2015 The changing role of ER in endocrine resistance. Breast 24 (Supplement 2) S60–S66. (10.1016/j.breast.2015.07.015) 26271713 PMC4666002

[bib93] Nettles KW Bruning JB Gil G Nowak J Sharma SK Hahm JB Kulp K Hochberg RB Zhou H Katzenellenbogen JA 2008 NFκB selectivity of estrogen receptor ligands revealed by comparative crystallographic analyses. Nature Chemical Biology 4 241–247. (10.1038/nchembio.76) 18344977 PMC2659626

[bib94] Nilsson S Makela S Treuter E Tujague M Thomsen J Andersson G Enmark E Pettersson K Warner M Gustafsson JA 2001 Mechanisms of estrogen action. Physiological Reviews 81 1535–1565. 11581496 10.1152/physrev.2001.81.4.1535

[bib95] Norris JD Fan D Stallcup MR McDonnell DP 1998 Enhancement of estrogen receptor transcriptional activity by the coactivator GRIP-1 highlights the role of activation function 2 in determining estrogen receptor pharmacology. Journal of Biological Chemistry 273 6679–6688. (10.1074/jbc.273.12.6679) 9506965

[bib96] O’Regan RM Jordan VC 2002 The evolution of tamoxifen therapy in breast cancer: selective oestrogen-receptor modulators and downregulators. Lancet Oncology 3 207–214. (10.1016/s1470-2045(02)00711-8) 12067682

[bib97] Onate SA Boonyaratanakornkit V Spencer TE Tsai SY Tsai MJ Edwards DP O’Malley BW 1998 The steroid receptor coactivator-1 contains multiple receptor interacting and activation domains that cooperatively enhance the activation function 1 (AF1) and AF2 domains of steroid receptors. Journal of Biological Chemistry 273 12101–12108. (10.1074/jbc.273.20.12101) 9575154

[bib98] Osborne CK Bardou V Hopp TA Chamness GC Hilsenbeck SG Fuqua SA Wong J Allred DC Clark GM Schiff R 2003 Role of the estrogen receptor coactivator AIB1 (SRC-3) and HER-2/neu in tamoxifen resistance in breast cancer. Journal of the National Cancer Institute 95 353–361. (10.1093/jnci/95.5.353) 12618500

[bib99] Osborne CK Coronado-Heinsohn EB Hilsenbeck SG McCue BL Wakeling AE McClelland RA Manning DL Nicholson RI 1995 Comparison of the effects of a pure steroidal antiestrogen with those of tamoxifen in a model of human breast cancer. Journal of the National Cancer Institute 87 746–750. (10.1093/jnci/87.10.746) 7563152

[bib100] Osborne CK Pippen J Jones SE Parker LM Ellis M Come S Gertler SZ May JT Burton G Dimery I 2002 Double-blind, randomized trial comparing the efficacy and tolerability of fulvestrant versus anastrozole in postmenopausal women with advanced breast cancer progressing on prior endocrine therapy: results of a North American trial. Journal of Clinical Oncology 20 3386–3395. (10.1200/JCO.2002.10.058) 12177098

[bib101] Pakdel F Reese JC Katzenellenbogen BS 1993 Identification of charged residues in an N-terminal portion of the hormone-binding domain of the human estrogen receptor important in transcriptional activity of the receptor. Molecular Endocrinology 7 1408–1417. (10.1210/me.7.11.1408) 8114756

[bib102] Palkowitz AD Glasebrook AL Thrasher KJ Hauser KL Short LL Phillips DL Muehl BS Sato M Shetler PK Cullinan GJ 1997 Discovery and synthesis of (6-hydroxy-3-(4-(2-(1-piperidinyl)ethoxy)phenoxy)-2-(4-hydroxyphenyl))b enzo[b]thiophene: a novel, highly potent, selective estrogen receptor modulator. Journal of Medicinal Chemistry 40 1407–1416. (10.1021/jm970167b) 9154963

[bib103] Park JB 2013 The effects of fulvestrant, an estrogen receptor antagonist, on the proliferation, differentiation and mineralization of osteoprecursor cells. Molecular Medicine Reports 7 555–558. (10.3892/mmr.2012.1200) 23179494

[bib104] Peekhaus NT Chang T Hayes EC Wilkinson HA Mitra SW Schaeffer JM Rohrer SP 2004 Distinct effects of the antiestrogen Faslodex on the stability of estrogen receptors-alpha and -beta in the breast cancer cell line MCF-7. Journal of Molecular Endocrinology 32 987–995. (10.1677/jme.0.0320987) 15171727

[bib105] Pike AC Brzozowski AM Hubbard RE Bonn T Thorsell AG Engstrom O Ljunggren J Gustafsson JA Carlquist M 1999 Structure of the ligand-binding domain of oestrogen receptor beta in the presence of a partial agonist and a full antagonist. EMBO Journal 18 4608–4618. (10.1093/emboj/18.17.4608) 10469641 PMC1171535

[bib106] Pike AC Brzozowski AM Walton J Hubbard RE Thorsell AG Li YL Gustafsson JA Carlquist M 2001 Structural insights into the mode of action of a pure antiestrogen. Structure 9 145–153. (10.1016/S0969-2126(01)00568-8) 11250199

[bib107] Rando G Horner D Biserni A Ramachandran B Caruso D Ciana P Komm B Maggi A 2010 An innovative method to classify SERMs based on the dynamics of estrogen receptor transcriptional activity in living animals. Molecular Endocrinology 24 735–744. (10.1210/me.2009-0514) 20197311 PMC2852355

[bib108] Reid G Hubner MR Metivier R Brand H Denger S Manu D Beaudouin J Ellenberg J Gannon F 2003 Cyclic, proteasome-mediated turnover of unliganded and liganded ERalpha on responsive promoters is an integral feature of estrogen signaling. Molecular Cell 11 695–707. (10.1016/S1097-2765(03)00090-X) 12667452

[bib109] Robertson JF Lindemann J Garnett S Anderson E Nicholson RI Kuter I Gee JM 2014 A good drug made better: the fulvestrant dose-response story. Clinical Breast Cancer 14 381–389. (10.1016/j.clbc.2014.06.005) 25457991

[bib110] Robinson DR Wu YM Vats P Su F Lonigro RJ Cao X Kalyana-Sundaram S Wang R Ning Y Hodges L 2013 Activating ESR1 mutations in hormone-resistant metastatic breast cancer. Nature Genetics 45 1446–1451. (10.1038/ng.2823) 24185510 PMC4009946

[bib111] Sanchez R Nguyen D Rocha W White JH Mader S 2002 Diversity in the mechanisms of gene regulation by estrogen receptors. BioEssays 24 244–254. (10.1002/bies.10066) 11891761

[bib112] Scafoglio C Ambrosino C Cicatiello L Altucci L Ardovino M Bontempo P Medici N Molinari AM Nebbioso A Facchiano A 2006 Comparative gene expression profiling reveals partially overlapping but distinct genomic actions of different antiestrogens in human breast cancer cells. Journal of Cellular Biochemistry 98 1163–1184. (10.1002/jcb.20820) 16514628

[bib113] Schafer JM Lee ES Dardes RC Bentrem D O’Regan RM De Los Reyes A Jordan VC 2001 Analysis of cross-resistance of the selective estrogen receptor modulators arzoxifene (LY353381) and LY117018 in tamoxifen-stimulated breast cancer xenografts. Clinical Cancer Research 7 2505–2512. 11489833

[bib114] Sefrioui D Perdrix A Sarafan-Vasseur N Dolfus C Dujon A Picquenot JM Delacour J Cornic M Bohers E Leheurteur M 2015 Short report: monitoring ESR1 mutations by circulating tumor DNA in aromatase inhibitor resistant metastatic breast cancer. International Journal of Cancer 137 2513–2519. (10.1002/ijc.29612) 25994408

[bib115] Seo HS Larsimont D Querton G El Khissiin A Laios I Legros N Leclercq G 1998 Estrogenic and anti-estrogenic regulation of estrogen receptor in MCF-7 breast-cancer cells: comparison of immunocytochemical data with biochemical measurements. International Journal of Cancer 78 760–765. (10.1002/(sici)1097-0215(19981209)78:6<760::aid-ijc14>3.0.co;2-u) 9833770

[bib116] Shang Y Brown M 2002 Molecular determinants for the tissue specificity of SERMs. Science 295 2465–2468. (10.1126/science.1068537) 11923541

[bib117] Shiau AK Barstad D Loria PM Cheng L Kushner PJ Agard DA Greene GL 1998 The structural basis of estrogen receptor/coactivator recognition and the antagonism of this interaction by tamoxifen. Cell 95 927–937. (10.1016/S0092-8674(00)81717-1) 9875847

[bib118] Shih HM Chang CC Kuo HY Lin DY 2007 Daxx mediates SUMO-dependent transcriptional control and subnuclear compartmentalization. Biochemical Society Transactions 35 1397–1400. (10.1042/BSbib351397) 18031230

[bib119] Smith CL Nawaz Z O’Malley BW 1997 Coactivator and corepressor regulation of the agonist/antagonist activity of the mixed antiestrogen, 4-hydroxytamoxifen. Molecular Endocrinology 11 657–666. (10.1210/mend.11.6.0009) 9171229

[bib120] Smith CL O’Malley BW 2004 Coregulator function: a key to understanding tissue specificity of selective receptor modulators. Endocrine Reviews 25 45–71. (10.1210/er.2003-0023) 14769827

[bib121] Su Q Hu S Gao H Ma R Yang Q Pan Z Wang T Li F 2008 Role of AIB1 for tamoxifen resistance in estrogen receptor-positive breast cancer cells. Oncology 75 159–168. (10.1159/000159267) 18827493

[bib122] Suh N Glasebrook AL Palkowitz AD Bryant HU Burris LL Starling JJ Pearce HL Williams C Peer C Wang Y 2001 Arzoxifene, a new selective estrogen receptor modulator for chemoprevention of experimental breast cancer. Cancer Research 61 8412–8415. 11731420

[bib123] Suresh PS Ma S Migliaccio A Chen G 2014 Protein-tyrosine phosphatase H1 increases breast cancer sensitivity to antiestrogens by dephosphorylating estrogen receptor at Tyr537. Molecular Cancer Therapeutics 13 230–238. (10.1158/1535-7163.MCT-13-0610) 24227889

[bib124] Tamm-Rosenstein K Simm J Suhorutshenko M Salumets A Metsis M 2013 Changes in the transcriptome of the human endometrial Ishikawa cancer cell line induced by estrogen, progesterone, tamoxifen, and mifepristone (RU486) as detected by RNA-sequencing. PLoS ONE 8 e68907 (10.1371/journal.pone.0068907) 23874806 PMC3712916

[bib125] Thomas RS Sarwar N Phoenix F Coombes RC Ali S 2008 Phosphorylation at serines 104 and 106 by Erk1/2 MAPK is important for estrogen receptor-alpha activity. Journal of Molecular Endocrinology 40 173–184. (10.1677/JME-07-0165) 18372406 PMC2277492

[bib126] Toy W Shen Y Won H Green B Sakr RA Will M Li Z Gala K Fanning S King TA 2013 ESR1 ligand-binding domain mutations in hormone-resistant breast cancer. Nature Genetics 45 1439–1445. (10.1038/ng.2822) 24185512 PMC3903423

[bib127] Turner RT Wakley GK Hannon KS Bell NH 1988 Tamoxifen inhibits osteoclast-mediated resorption of trabecular bone in ovarian hormone-deficient rats. Endocrinology 122 1146–1150. (10.1210/endo-122-3-1146) 3342747

[bib128] Tzukerman MT Esty A Santiso-Mere D Danielian P Parker MG Stein RB Pike JW McDonnell DP 1994 Human estrogen receptor transactivational capacity is determined by both cellular and promoter context and mediated by two functionally distinct intramolecular regions. Molecular Endocrinology 8 21–30. (10.1210/me.8.1.21) 8152428

[bib129] Van de Velde P Nique F Bouchoux F Bremaud J Hameau MC Lucas D Moratille C Viet S Philibert D Teutsch G 1994 RU 58,668, a new pure antiestrogen inducing a regression of human mammary carcinoma implanted in nude mice. Journal of Steroid Biochemistry and Molecular Biology 48 187–196. (10.1016/0960-0760(94)90144-9) 8142294

[bib130] Varlakhanova N Snyder C Jose S Hahm JB Privalsky ML 2010 Estrogen receptors recruit SMRT and N-CoR corepressors through newly recognized contacts between the corepressor N terminus and the receptor DNA binding domain. Molecular and Cellular Biology 30 1434–1445. (10.1128/MCB.01002-09) 20065040 PMC2832498

[bib131] Vogel VG Costantino JP Wickerham DL Cronin WM Cecchini RS Atkins JN Bevers TB Fehrenbacher L Pajon ER Wade JL 3rd 2010 Update of the national surgical adjuvant breast and bowel project study of tamoxifen and raloxifene (STAR) P-2 trial: preventing breast cancer. Cancer Prevention Research 3 696–706. (10.1158/1940-6207.CAPR-10-0076) 20404000 PMC2935331

[bib132] Wakeling AE 1993 The future of new pure antiestrogens in clinical breast cancer. Breast Cancer Research and Treatment 25 1–9. (10.1007/BF00662395) 8518404

[bib133] Wakeling AE Dukes M Bowler J 1991 A potent specific pure antiestrogen with clinical potential. Cancer Research 51 3867–3873. 1855205

[bib134] Ward RL Morgan G Dalley D Kelly PJ 1993 Tamoxifen reduces bone turnover and prevents lumbar spine and proximal femoral bone loss in early postmenopausal women. Bone and Mineral 22 87–94. (10.1016/S0169-6009(08)80220-6) 8251768

[bib135] Wardell SE Kazmin D McDonnell DP 2012 Research resource: transcriptional profiling in a cellular model of breast cancer reveals functional and mechanistic differences between clinically relevant SERM and between SERM/estrogen complexes. Molecular Endocrinology 26 1235–1248. (10.1210/me.2012-1031) 22570330 PMC3385791

[bib136] Wardell SE Marks JR McDonnell DP 2011 The turnover of estrogen receptor alpha by the selective estrogen receptor degrader (SERD) fulvestrant is a saturable process that is not required for antagonist efficacy. Biochemical Pharmacology 82 122–130. (10.1016/j.bcp.2011.03.031) 21501600 PMC3109090

[bib137] Wardell SE Nelson ER Chao CA McDonnell DP 2013 Bazedoxifene exhibits antiestrogenic activity in animal models of tamoxifen-resistant breast cancer: implications for treatment of advanced disease. Clinical Cancer Research 19 2420–2431. (10.1158/1078-0432.CCR-12-3771) 23536434 PMC3643989

[bib138] Warnmark A Treuter E Gustafsson JA Hubbard RE Brzozowski AM Pike AC 2002 Interaction of transcriptional intermediary factor 2 nuclear receptor box peptides with the coactivator binding site of estrogen receptor alpha. Journal of Biological Chemistry 277 21862–21868. (10.1074/jbc.M200764200) 11937504

[bib139] Weatherman RV Fletterick RJ Scanlan TS 1999 Nuclear-receptor ligands and ligand-binding domains. Annual Review of Biochemistry 68 559–581. (10.1146/annurev.biochem.68.1.559) 10872460

[bib140] Webb P Nguyen P Kushner PJ 2003 Differential SERM effects on corepressor binding dictate ERalpha activity in vivo. Journal of Biological Chemistry 278 6912–6920. (10.1074/jbc.M208501200) 12482846

[bib141] Webb P Nguyen P Shinsako J Anderson C Feng W Nguyen MP Chen D Huang SM Subramanian S McKinerney E 1998 Estrogen receptor activation function 1 works by binding p160 coactivator proteins. Molecular Endocrinology 12 1605–1618. (10.1210/mend.12.10.0185) 9773983

[bib142] Weis KE Ekena K Thomas JA Lazennec G Katzenellenbogen BS 1996 Constitutively active human estrogen receptors containing amino acid substitutions for tyrosine 537 in the receptor protein. Molecular Endocrinology 10 1388–1398. (10.1210/me.10.11.1388) 8923465

[bib143] Welboren WJ van Driel MA Janssen-Megens EM van Heeringen SJ Sweep FC Span PN Stunnenberg HG 2009 ChIP-Seq of ERalpha and RNA polymerase II defines genes differentially responding to ligands. EMBO Journal 28 1418–1428. (10.1038/emboj.2009.88) 19339991 PMC2688537

[bib144] White JH Fernandes I Mader S Yang XJ 2004 Corepressor recruitment by agonist-bound nuclear receptors. Vitamins and Hormones 68 123–143. (10.1016/s0083-6729(04)68004-6) 15193453

[bib145] Wijayaratne AL McDonnell DP 2001 The human estrogen receptor-alpha is a ubiquitinated protein whose stability is affected differentially by agonists, antagonists, and selective estrogen receptor modulators. Journal of Biological Chemistry 276 35684–35692. (10.1074/jbc.M101097200) 11473106

[bib146] Wijayaratne AL Nagel SC Paige LA Christensen DJ Norris JD Fowlkes DM McDonnell DP 1999 Comparative analyses of mechanistic differences among antiestrogens. Endocrinology 140 5828–5840. (10.1210/en.140.12.5828) 10579349

[bib147] Wittmann BM Sherk A McDonnell DP 2007 Definition of functionally important mechanistic differences among selective estrogen receptor down-regulators. Cancer Research 67 9549–9560. (10.1158/0008-5472.CAN-07-1590) 17909066

[bib148] Wu YL Yang X Ren Z McDonnell DP Norris JD Willson TM Greene GL 2005 Structural basis for an unexpected mode of SERM-mediated ER antagonism. Molecular Cell 18 413–424. (10.1016/j.molcel.2005.04.014) 15893725

[bib149] Zhang QX Borg A Wolf DM Oesterreich S Fuqua S 1997 An estrogen receptor mutant with strong hormone-independent activity from a metastatic breast cancer. Cancer Research 57 1244–1249. 9102207

[bib150] Zwart W de Leeuw R Rondaij M Neefjes J Mancini MA Michalides R 2010 The hinge region of the human estrogen receptor determines functional synergy between AF-1 and AF-2 in the quantitative response to estradiol and tamoxifen. Journal of Cell Science 123 1253–1261. (10.1242/jcs.061135) 20332105

